# Mariner: explore the Hi-Cs

**DOI:** 10.1093/bioinformatics/btae352

**Published:** 2024-05-30

**Authors:** Eric S Davis, Sarah M Parker, Nicole E Kramer, J P Flores, Manjari Kiran, Douglas H Phanstiel

**Affiliations:** Curriculum in Bioinformatics and Computational Biology, University of North Carolina at Chapel Hill, Chapel Hill, NC, 27599, United States; Thurston Arthritis Research Center, University of North Carolina at Chapel Hill, Chapel Hill, NC, 27599, United States; Curriculum in Bioinformatics and Computational Biology, University of North Carolina at Chapel Hill, Chapel Hill, NC, 27599, United States; Curriculum in Bioinformatics and Computational Biology, University of North Carolina at Chapel Hill, Chapel Hill, NC, 27599, United States; Curriculum in Bioinformatics and Computational Biology, University of North Carolina at Chapel Hill, Chapel Hill, NC, 27599, United States; Department of Systems and Computational Biology, School of Life Sciences, University of Hyderabad, Hyderabad, Telangana 500046, India; Curriculum in Bioinformatics and Computational Biology, University of North Carolina at Chapel Hill, Chapel Hill, NC, 27599, United States; Thurston Arthritis Research Center, University of North Carolina at Chapel Hill, Chapel Hill, NC, 27599, United States; Department of Cell Biology & Physiology, University of North Carolina at Chapel Hill, Chapel Hill, NC, 27599, United States; Lineberger Comprehensive Cancer Center, University of North Carolina at Chapel Hill, Chapel Hill, NC, 27599, United States; Curriculum in Genetics & Molecular Biology, University of North Carolina at Chapel Hill, Chapel Hill, NC, 27599, United States

## Abstract

**Motivation:**

3D chromatin structure plays an important role in regulating gene expression and alterations to this structure can result in developmental abnormalities and disease. While genomic approaches like Hi-C and Micro-C can provide valuable insights in 3D chromatin architecture, the resulting datasets are extremely large and difficult to manipulate.

**Results:**

Here, we present *mariner*, a rapid and memory efficient tool to extract, aggregate, and plot data from Hi-C matrices within the R/Bioconductor environment. *Mariner* simplifies the process of querying and extracting contacts from multiple Hi-C files using a parallel and block-processing approach. Modular functions allow complete workflow customization for advanced users, yet all-in-one functions are available for running the most common types of analyses. Finally, tight integration with existing Bioconductor infrastructure enables complete analysis and visualization of Hi-C data in R.

**Availability and implementation:**

Available on GitHub at https://github.com/EricSDavis/mariner and on Bioconductor at https://www.bioconductor.org/packages/release/bioc/html/mariner.html.

## 1 Introduction

3D genome organization plays an important role in the regulation of gene expression during human development and disease. Chromatin features like loops, topologically associating domains (TADs), and compartments bring actively transcribed regions into proximity with their linearly distant regulators. Multiple genomic assays—including Hi-C, Micro-C, and HiChIP—have been created to study chromatin structure. These techniques have been accompanied by the development of associated tools for basic processing and feature identification (Servant *et al.* 2015, Durand *et al.* 2016); however, extracting biological insights from the resulting datasets requires versatile software for data extraction, manipulation, and visualization. Tools for querying, extracting, and aggregating 3D chromatin contacts are continuing to emerge (Flyamer *et al.* 2020, Sahin *et al.* 2021, Abdennur *et al.* 2022, Chang *et al.* 2022) but there is still a need for flexible, memory efficient, and easy-to-use tools to manipulate 3D chromatin data that integrate well into existing computational ecosystems.

Here, we introduce *mariner*, a complete suite of tools for exploring Hi-C data in R. An overview of *mariner* functionality is depicted in Supplementary Fig. S1. *mariner* combines existing and novel functionality into an efficient and easy to use Bioconductor package. Bioconductor’s infrastructure of classes for genomic data types allows interoperability between software packages (Gentleman *et al.* 2004). *Mariner* extends this infrastructure with classes and methods for efficiently storing and operating on Hi-C submatrices directly in R. These tools are flexible and modular, enabling full customization of analyses and facilitating extension by future developers. *Mariner* forms a software ecosystem with several existing Bioconductor packages enabling the analysis and visualization of Hi-C data without leaving R. Detailed publicly available vignettes and workshops demonstrate how to create workflows for conducting differential analysis and data aggregation. Together, these tools will empower biologists to explore Hi-C data to better understand the interplay between 3D chromatin structure and gene regulation.

## 2 Key features

*Mariner* can extract data from .hic and .(m)cool files with speed and flexibility (Supplementary Fig. S1). The *pullHicPixels* function allows users to extract raw or normalized counts for a list of genomic interactions. Counts can be simultaneously pulled across a list of .hic files or .(m)cool facilitating easy comparison across samples for analyses including differential loop detection. The *pullHicMatrices* function pulls contacts corresponding to submatrices (e.g. contact domains, stripes, or loops with surrounding pixels). *pullHicMatrices* allows extraction of both regular and irregularly sized matrices from multiple Hi-C files. Both functions use a block-processing approach (Morgan *et al.* 2023) which provides high speed performance without overwhelming working memory. Users can fine-tune the amount of contacts that are read and processed at a time by defining the block size or number of blocks; however, we encourage users to employ default parameters unless they encounter memory exceptions. A detailed evaluation of the speed of extraction at various block sizes, and in comparison to the existing tool strawr, is provided in Supplementary Fig. S3A.

Extracted data is stored in a memory efficient but accessible object. Blocks are stored on-disk in an HDF5 file, which enables fast, random access to any portion of the data for downstream functions without overwhelming working memory. Accessor functions including counts, interactions, and metadata allow users to easily retrieve and manipulate stored data. *Mariner* extends the *DelayedArray*, *HDF5Array*, and *InteractionSet* packages, creating an interface that handles this complexity so the data appears to be stored in working memory (Lun *et al.* 2016, Pagès 2020, Pagès *et al.* 2021). The HDF5-based data objects that mariner uses—InteractionMatrix, InteractionArray, and InteractionJaggedArray—occupy far less memory than traditional R objects such as data frames (Supplementary Fig. S3B).

*Mariner* allows highly flexible aggregation of extracted data (Supplementary Fig. S2). Performing pileup analyses on loops, domains, or boundary sites are key methods for assessing genome-wide trends in contact frequency. *Mariner* enables completely customizable aggregation of 3D chromatin data. All-in-one functions (i.e. *pileupPixels*, *pileupDomains*, *pileupBoundaries*) are provided for creating aggregate peak analysis (APA) plots, aggregate TAD plots, and saddle plots at boundary regions. Alternatively, dedicated functions for extracting and aggregating contacts (e.g. *pullHicMatrices*, *removeShortPairs*, *regularize*, *aggHicMatrices*) can be used independently to aggregate across interactions, datasets, or both. This maximizes flexibility while providing out-of-box solutions for common analyses.

*Mariner* provides new methods for characterizing chromatin loops (pixels that exhibit significant enrichment to local background), which play an important role in shaping the regulatory landscape. The stochastic nature of data collection often results in the same loop being assigned to slightly different pixels across datasets. The *mergePairs* function identifies these redundant loops using the DBSCAN algorithm (Hahsler *et al.* 2019) and assigns them to a representative pixel based on frequency of detection, number of counts, or any other user-defined metric (Supplementary Fig. S3). This is a critical step that increases power and accuracy of differential loop analyses. Importantly, *mariner* includes a new *MergedGInteractions* class object to store the data which retains all of the metadata of the unmerged loops. *Mariner* also includes the *calcLoopEnrichment* function that can calculate the enrichment of any pixel compared to a customizable local background (Supplementary Fig. S4). This provides a metric of “loop strength” which is critical for a number of downstream analyses. For example, this metric is often used to select the representative pixel for merged pairs.

Finally, visualization is a key aspect to Hi-C analysis. *Mariner* forms a unified ecosystem with the *plotgardener* Bioconductor package for creating publication-quality, multi-panel genomic figures (Kramer *et al.* 2022). Together, these packages allow users to perform Hi-C analysis and visualize the results directly in R without the need of external software.

## 3 Conclusion

*Mariner* provides a fast and memory efficient solution for performing Hi-C analysis in R. Using a parallel and block-processing approach, *mariner* simplifies the process of querying and extracting contacts from multiple Hi-C files across a set of shared interactions. Modular functions and wrappers for common workflows provide a comprehensive, yet flexible suite of tools for working with contact data. Finally, the class structure of *mariner* is highly integrated with existing Bioconductor packages resulting in a cohesive and unified ecosystem for Hi-C analysis in R. *Mariner* is available through Bioconductor (https://www.bioconductor.org/packages/release/bioc/html/mariner.html) with the latest development version on GitHub (https://github.com/EricSDavis/mariner). Full documentation and vignettes are available at https://ericscottdavis.com/mariner.

## Supplementary Material

btae352_Supplementary_Data
