# Supplementary material for: Mariner: explore the Hi-Cs
Source: Bioinformatics. 2024 May 30;40(6):btae352. doi: 10.1093/bioinformatics/btae352 (PMC11176088; doi:10.1093/bioinformatics/btae352)
Supplement: btae352_Supplementary_Data [file btae352_supplementary_data.pdf]

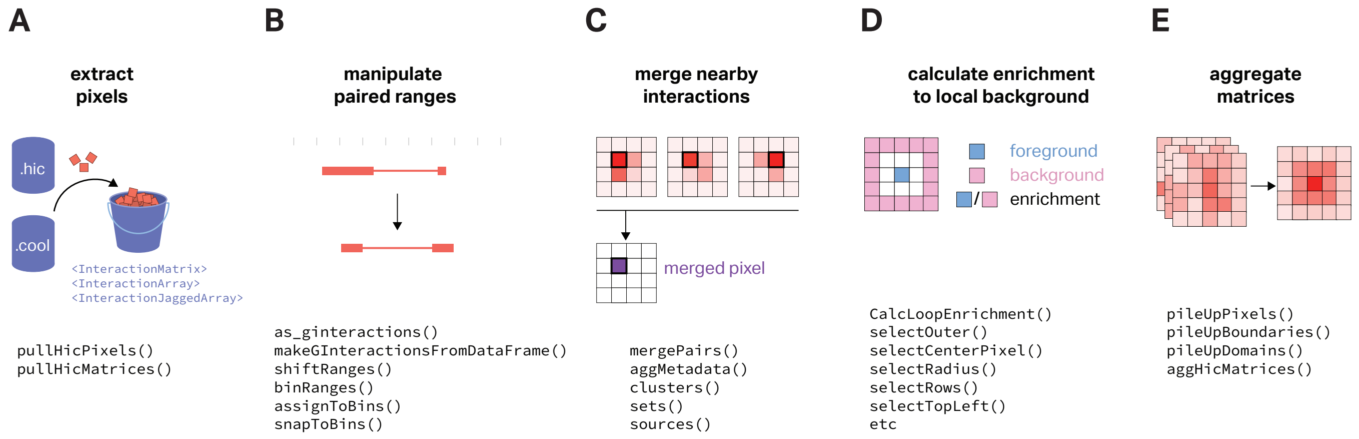

Supplemental Figure 1. Overview of key features. *Mariner* functions can be grouped into five broad categories. *Mariner* can **(A)** extract pixels, **(B)** manipulate paired ranges, **(C)** merge nearby interactions, **(D)** calculate enrichment to local background, and **(E)** aggregate matrices.

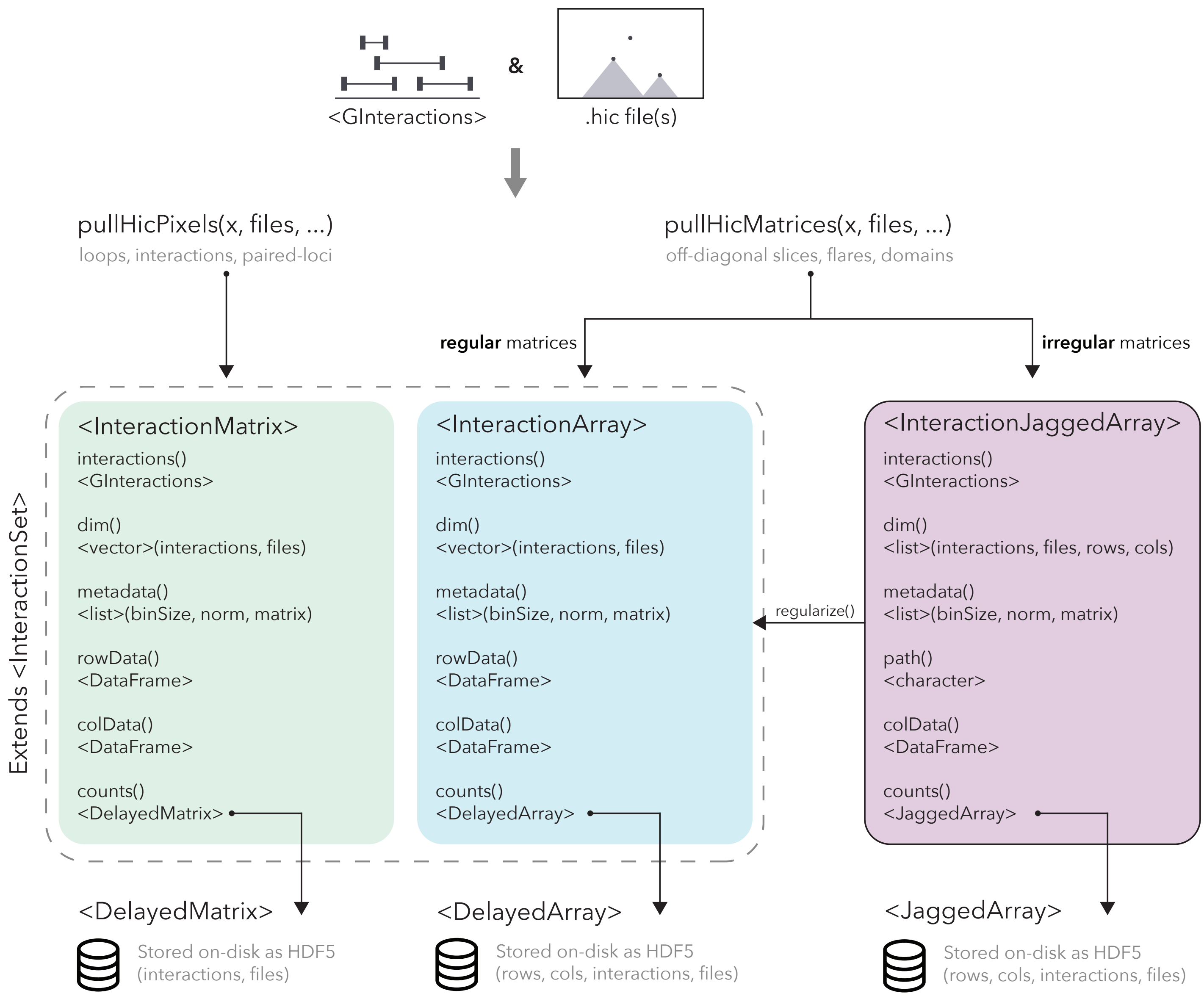

Supplemental Figure 2. Efficient extraction of Hi-C pixels and submatrices. Diagram showing the two major Hi-C extraction functions for pulling pixels (*pullHicPixels* and *pullHicMatrices*) and regular or irregular submatrices they return given a set of interactions as a GInteractions object and one or more ".hic" files. Extraction results in one of three classes, *InteractionMatrix*, *InteractionArray*, or *InteractionJaggedArray*, that hold metadata associated with each interaction and ".hic" file as well as count data that is stored on-disk as an HDF5 object.

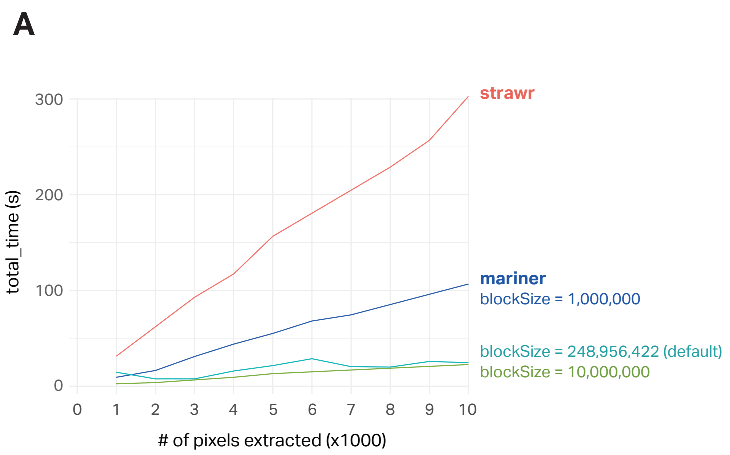

Supplemental Figure 3. Benchmarking speed and memory usage. (A) A line plot representing the total time required for extraction of pixels by strawr or mariner using three different values of block size. **(B)** A line plot representing the memory used to store extracted pixels either as a data frame or the mariner object InteractionArray.

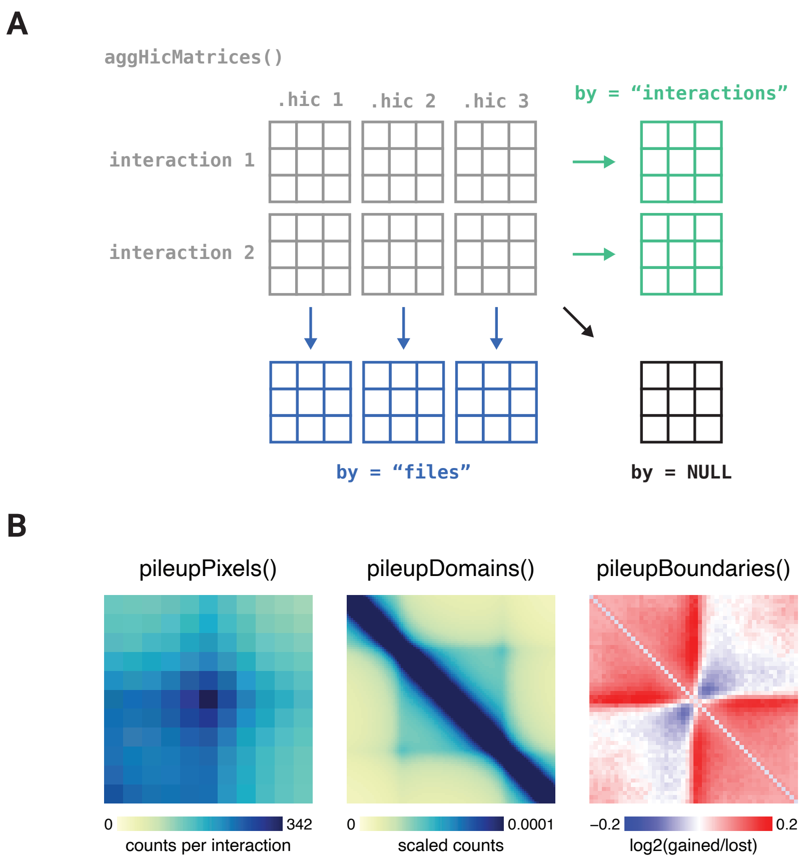

Supplemental Figure 4. Flexible aggregation of Hi-C feature matrices. (A) Diagram demonstrating how the *aggHicMatrices* function can be used to flexibly aggregate extracted Hi-C submatrices by interactions, Hi-C files, or both. **(B)** *Mariner* includes all-in-one functions for performing common Hi-C aggregation analyses such as pileup analysis of loops (*pileupPixels*), aggregation of domains (*pileupDomains*), and saddle plots at boundary regions (*pileupBoundaries*). These functions can be used in combination with the *plotMatrix* function to visualize the results.

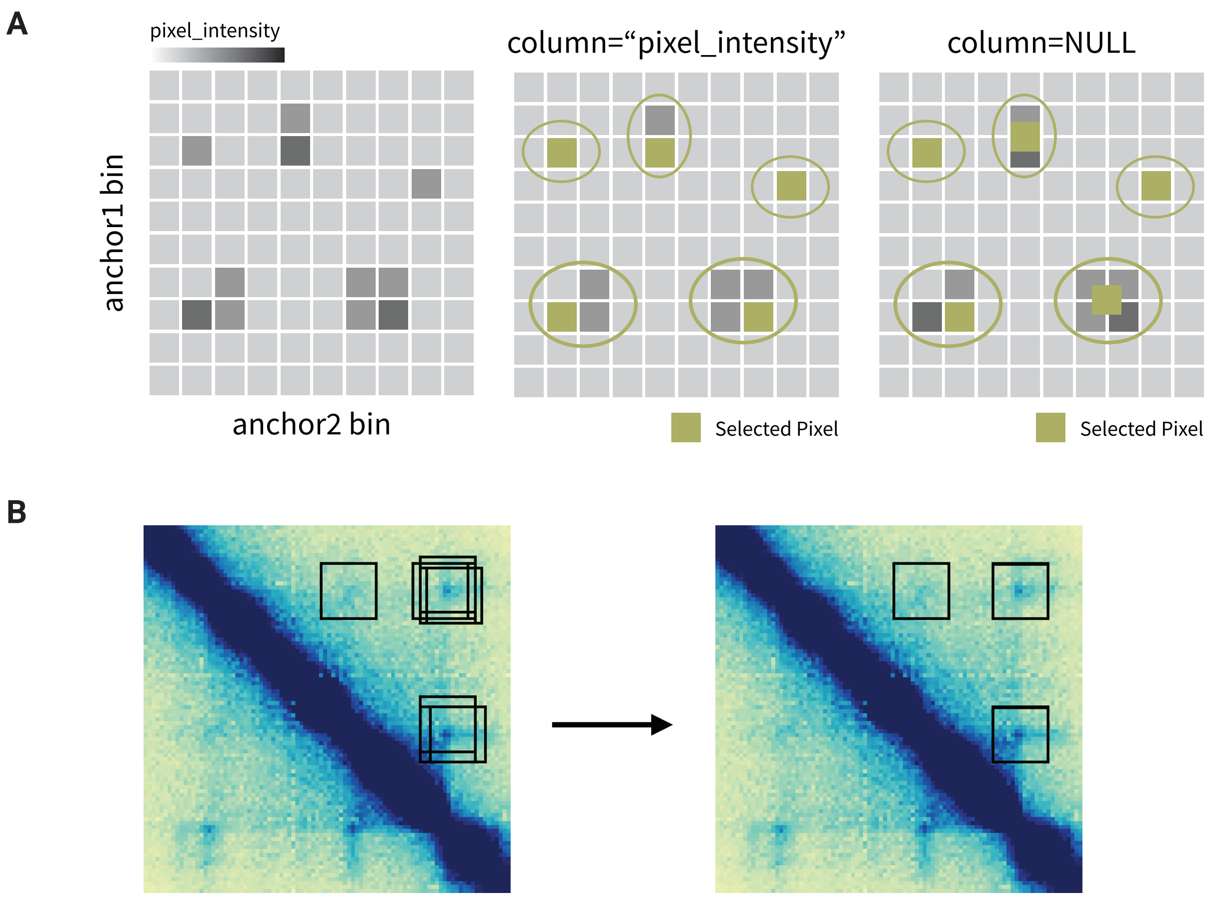

Supplemental Figure 5. The *mergePairs* function resolves redundant loop calls. Stochasticity in data collection results in redundant loop calls across replicates or conditions and can decrease the power to detect differences in loops between treatment conditions. *Mariner* solves this problem with the mergePairs function which uses DBSCAN to cluster redundant loop anchors, and assigns them to a representative pixel based on frequency of detection, number of counts, or any other user-defined metric. **(A)** Diagram illustrating how pixels are selected when a user supplies a metric (in this case pixel intensity) to the "column" argument of *mergePairs* (center diagram). When no metric is supplied ("column=NULL"), the selected interaction is the median of modes among anchors in each cluster. **(B)** An example of loop calls before and after mergePairs.

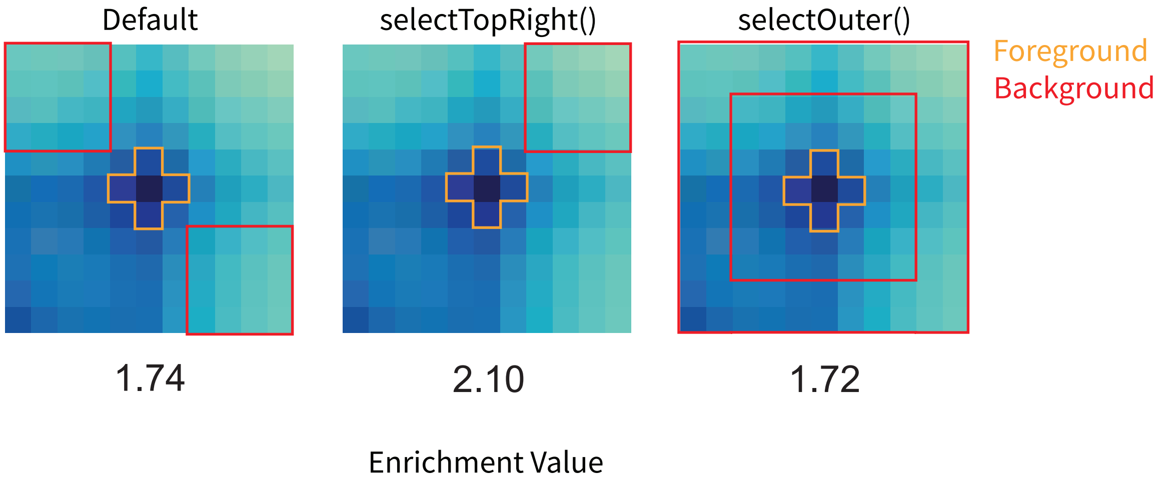

Supplemental Figure 6. Scoring pixel enrichment to local background. *Mariner* provides the calcLoopEnrichment function for determining the enrichment of a pixel (or set of pixels) to their local background. There are 14 included functions for selecting the foreground and background that can be combined to produce a customizable selection. The function also supports user-defined enrichment functions for custom enrichment calculation.
